# Cardiac surgery in East Africa: a profile of cases and referral to physiotherapy

**DOI:** 10.4314/ahs.v23i2.37

**Published:** 2023-06

**Authors:** Abdallah R Makalla, Farhana Karachi, Joliana S Phillips

**Affiliations:** 1 The Department of Rehabilitation Medicine Muhimbili National Hospital P.O.Box 65000, Dar Es Salaam, Tanzania; 2 Department of Physiotherapy, University of the Western Cape, Private Bag X17 Bellville 7535

**Keywords:** Profile, cardiac surgery, cardiac disease, hospital stay, physiotherapy referral

## Abstract

**Background:**

A significant increase in cardiac surgery has been observed globally, with prolonged length of stay (LOS) still prevalent due to post-operative complications. Physiotherapy pre and post cardiac surgery is known to reduce these complications and LOS, however cases referred for physiotherapy is unknown.

**Objective:**

The study aimed to describe the profile and pre- and post-operative referrals to physiotherapy of patients admitted to the cardiothoracic unit at a selected hospital in Tanzania over a four-year period.

**Method:**

Descriptive, retrospective design. A data extraction sheet was used to capture demographic, cardiac disease, ICU and hospital LOS, post-operative complications and physiotherapy referral data of all patients ≥18 years of age.

**Results:**

105 cardiac surgeries were performed. Patients' mean age was 30.6 years (SD=10.48) and 54.3% (n=57/105) were female. Cardiac surgeries performed declined from 48.6% (n=51/105) in 2010 to 10.5% (n=11/105) in 2013. Cardiac arrest (33%, n=7/21), pneumonia (19%, n=4/21) and lung collapse (4.8%, n=1/21) were the notable documented post-operative complications. ICU mortality was highest (72,7%, n=8/11). Only 1% (n=1/105) of cases were referred pre-operatively versus 77.7% (n=80/103) post-operatively for physiotherapy.

**Conclusion:**

Cardiac surgeries were reduced annually but the post-operative complications need to be reduced. Pre-operative physiotherapy referral may reduce pneumonia and lung collapse following cardiac surgery.

## Introduction

Since the introduction of cardiac surgery in the late 1950s, the number of cardiac procedures has increased significantly^1^. Indeed, the leading diseases in the Global Burden of Disease 2010, showed that cardiovascular and circulatory diseases were the leading diseases (11.8%), with the majority of these diseases amenable to surgical intervention^2^. In 2019, Vervoort, Meuris, Meyns and Verbrugghe^3^ also confirmed that the burden of cardiovascular disease (CVD) is increasing and is the leading cause of death globally^3^. Vervoort et al.^3^ states that CVD accounts for approximately 17.5 million deaths globally every year, of which 80% occurs in low and middle-income countries. Therefore, a significant increase in the number of patients with cardiovascular diseases requiring cardiac surgery has been observed^3^ globally and specifically in these low and middle-income countries^3^,^4^. There is only one cardiac surgery centre per 38 million inhabitants in sub-Saharan Africa, excluding South Africa, with only one cardiothoracic surgeon per 4 million people, accounting for only 1% of that total global capacity^3^. Vervoort et al.^3^ reported that “on a population level, ready access to cardiothoracic surgery is proportional to the economic status of the patient” and this results in approximately 4.5 billion people that do not have access to cardiac surgery^3^. In Africa, cardiovascular disease account for 9.2% of total deaths occurring on the continent annually, with reported hospital mortality reaching 9.2% in Cameroon and 21.9% in Tanzania^5^. Tanzania experienced a rapid increase in cardiovascular diseases, approximately 26% annually^6^.

Unlike other types of surgery, cardiac surgeries are expensive, demanding well-organized facilities equipped with the necessary equipment and qualified experts. Although there has been a variation of cardiac surgery data widely as some countries do not include private hospitals, while others do^7^; it has been reported that 234 million major cardiac surgery procedures are performed globally per annum^8^. Almost 73.6% of these cardiac surgeries were performed in developed countries which consists of 30% of the world's population. This indicates that 70% of the world population living in low and middle-income countries do not have access to this service^3^. Countries like Brazil, which is among the developing countries, perform about 70 000 cardiovascular procedures annually and almost 20 000 pacemaker implantations^9^. In contrast to this, seven procedures were performed in Zambia with the assistance of visiting cardiac surgeons in 2011^10^, while Nigeria, reported 51 cardiac surgeries performed for a period of eight years at the Lagos University Teaching Hospital^11^. In Tanzania, 105 procedures were reported over a period of one year from May 2008 to June 2009^12^.

In Tanzania, the plans to establish a cardiac unit started in the early 1970s but could not mature till 2005. In 2005, a strong commitment by the government was taken to establish this cardiac unit that included sending the team of staff to train in various institutions^12^. This team composed all various cadres of staff required to make a complete cardiac team. Further commitment was also necessary to acquire the equipment and tools that was managed successfully^12^. In 2007, appropriate measures were taken to officially start open heart surgery that started as of the 21^st^ May 2008. By June 2009 a total of 105 cases had been operated at Muhimbili National Hospital in Dar es Salaam, Tanzania^12^.

In the case of Tanzania, the country has been incurring high costs by sending patients with cardiovascular related complications to India for cardiac surgeries and other related treatment. In the 2011/12 financial year, the Tanzanian Ministry of Health and Social Welfare (MoHSW) was expected to spend 13 billion shillings for treatment of cardiac patients outside the country^6^. This accounted for 64.9% of all patients who were referred abroad for treatment in 2011/ 2012, a huge burden on the country's economy. As a result, the government was finalising construction of a complex for cardiac surgery at Muhimbili National Hospital (MNH). The complex was considered as a part of the long-term measures undertaken to build national capacity in taking care of cardiovascular cases in Tanzania^6^.

The management of patients undergoing cardiac surgery requires a multidisciplinary team approach in all phases of pre- and post-operative care. In the process of managing patients, there are numerous complications which may arise, such as pulmonary complications, bad posture and paralysis which could require the intervention of a physiotherapist. However, the position of physiotherapists in the prevention or reduction of these complications with other cardiac multidisciplinary team members is not well defined, as noted by Moreno et al.^13^. This could be due to a lack of clarity on the role of physiotherapy in this field of study, which leads to team members questioning the necessity of having physiotherapists in the cardiac team^14^. This ambiguity is also present in Tanzania; as other multidisciplinary team members concur with McAuley^15^ who argue that physiotherapy is only beneficial when offered to the at-risk patients who are prone to develop chest complications. On the other hand, others, Miranda et al.^16^ argue for the necessity of physiotherapy, stressing the fact that it is essential in pre- and post-operative phases of cardiac surgery.

While the discussion persists, cardiac surgery continues and patients are prone to develop complications. Physical and pulmonary complications namely poor posture, hemiparesis, atelectasis, impaired gaseous exchange and reduced functional residual capacity of the lung are among the post-operative complications^13^,^17^,^18^. For example, the incidence of atelectasis increases following anaesthesia during surgery which leads to pulmonary complication^19^. In Tanzania, these post-operative complications following cardiac surgery were and are still a cause of prolonged hospitalisation of up to 50 days^12^. To grapple with these complications, the literature suggests early and effective post-operative multidisciplinary team intervention involving physiotherapists.^14^ In Tanzania, physiotherapists are allowed to act as first contact/autonomous practitioners^20^ however, there is still a reported dependence on the referrals from Cardiothoracic Surgeons that can influence early intervention^21^. However, in Tanzania, to date only one study by Nyawawa et al.^12^, has described the frequency and type of cardiac surgeries since the development of the cardiac unit at Muhimbili National Hospital (MNH) in 2007 and the start of the first cardiac surgery procedure in May 2008 and the related complications post-surgery in one year.

Therefore, the aim of this paper is to describe the profile of the patients admitted to the cardiothoracic unit of MNH in Dar es Salaam, Tanzania from 1 January 2010 to 31^st^ December 2013 and their referrals to physiotherapy both pre-operatively and post-operatively.

## Methods

### Selection and Description of Participants

The study was conducted at MNH, which is the largest referral and teaching hospital in Dar es Salaam, Tanzania. The study sample included all patients ≥18years of age who underwent cardiac surgery from 1 January 2010 to 31 December 2013. Post-operative patients transferred from other cardiac units, both within and outside of Tanzania were excluded from the study. At the time the study was conducted MNH was the only hospital in Tanzania with a cardiac unit performing cardiac surgeries and thus the focus or interest of this study was only regarding the cardiac surgery cases and care processes of patients admitted directly to and operated on at MNH providing a baseline for cardiac surgery in Tanzania.

### Technical Information

Ethics approval to conduct the study was obtained from the Research Ethics Committee of the University of the Western Cape (Ethics Registration Number:14/2/7) and the management of MNH (Research Clearance Number: 545 2013/2014). Written informed consent was granted by the Head of the Cardiovascular Medicine Department to access the data. Data was collected using a data extraction sheet previously developed^12^ and modified for this study. A section that captured physiotherapy care and content was added to the original data extraction sheet. The first part of the data extraction sheet captured socio-demographic information such as age, gender, type of surgery, and diagnosis as confirmed by electrocardiogram (ECG). The second part captured clinical related information such as the number of days in hospital, namely pre-operative, ICU and post-operative periods separately. The third section captured the outcomes of management, namely complications, discharge information and mortality. The last section captured information with regards to physiotherapy referral numbers pre- and post-operatively.

The data extraction sheet was piloted for content validity and reliability and to test and establish the time accuracy of the data sheet in all four sections and to determine if any improvement was needed before applying it. The content was valid and was found to cover the appropriate and necessary information. The data extraction sheet was found to be reliable and took 15 – 20 minutes to complete for one patient record. Data from all patient records from patients who were admitted during 1 January 2010 to 31 December 2013, and fit the inclusion criteria were examined and captured in the data extraction sheet by the researcher and trained research assistant (a qualified physiotherapist with good knowledge of medical terminology and the data extraction process). Data from the data extraction sheet was entered into a Microsoft Excel Spreadsheet and checked and verified for accuracy. The excel was imported into SPSS version 22 for statistical analysis.

### Statistics

Descriptive statistics was used to summarize the socio-de mographic data, characteristics of cardiac surgery, duration of hospital stay (i.e., pre-, ICU and post-operative stay), outcome of management and physiotherapy number of referrals pre- and post-operatively. Data was analysed using the Statistical Package for Social Sciences (SPSS) version 22 and presented using frequency tables and figures. Data was expressed in means, standard deviations and percentages.

## Results

Medical records of a total of 105 adult (≥18years) cardiac cases who underwent cardiac surgery between 1 January 2010 to 31 December 2013 were reviewed.

### The profile of cardiac surgery cases

More than half (54.3%, n= 57/105) of the sample were females and the mean age of the sample were 30.6 years (SD=10.48).

**Figure 1:**
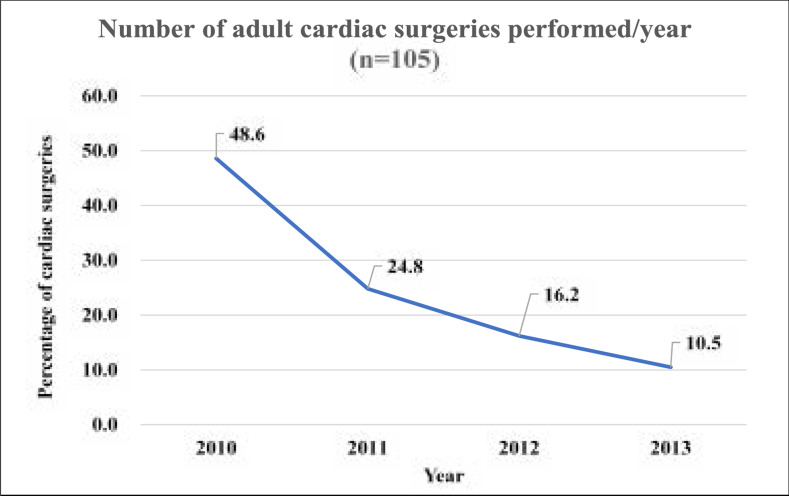
Distribution of the number of adult cardiac surgeries performed/year (n=105)

Figure 1 demonstrates the annual decline of cardiac surgery procedures performed at the hospital. In 2010, 48.6% (n=51/105) had cardiac surgery. This number decreased yearly to 10.5% (n=11/105) by 2013.

**Figure 2:**
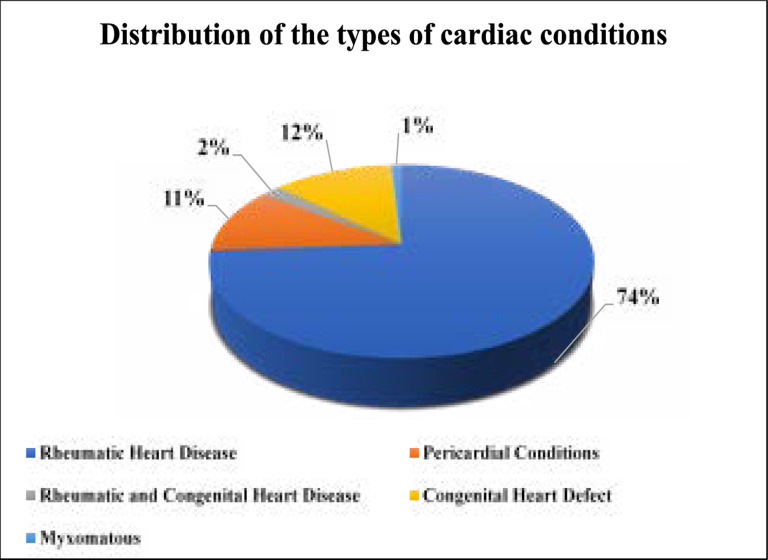
Distribution of the types of cardiac conditions

Rheumatic heart disease accounted for the majority of cases that underwent cardiac surgery (74.3%, n=78/105) followed by congenital heart disease (12%, n=13/105) and pericardial conditions 11% (n=12/105) as illustrated in Figure 2. The majority of cardiac procedures conducted in the four-year period at MNH included mitral valve involvement (71.4%, n=75/105) with or without any other heart valve repair. Pre-operative diagnosis confirmed by ECG, showed that a combination of mitral valve disease plus tricuspid valve regurgitation accounted for one third (32.4%, n=34/105) of the cases, a combination of mitral valve disease plus atrial valve involvement accounted for 6.7% (n=7/105), while mitral valve without a combination of any other valve disease accounted for (29.5%, n=31/105). Almost 42% (n=44/105) of all cardiac patients operated at the hospital had double valve replacement or repair.

**Figure 3:**
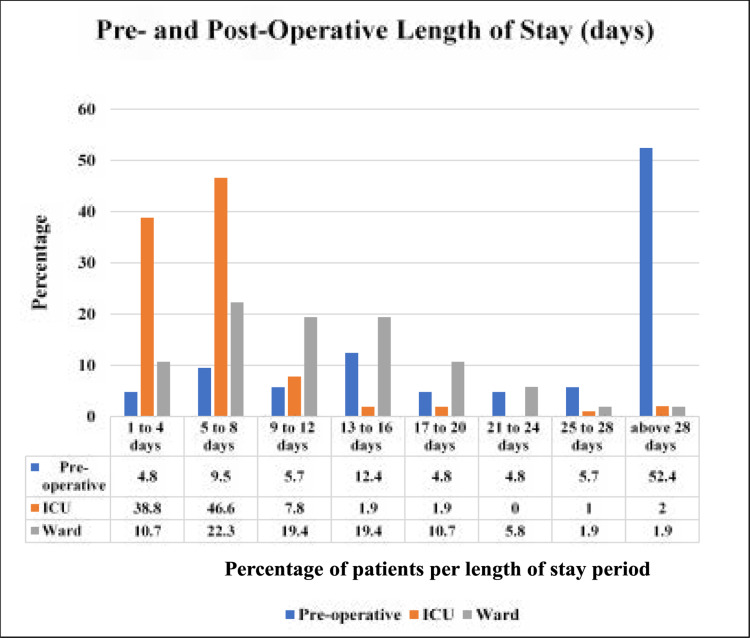
Pre- and Post-Operative Length of Stay (days)

The number of days stayed in hospital was captured and divided into pre-operative and post-operative length of stay (LOS) in days. Pre-operative stay was calculated from the day of admission to the day of surgery. The mean LOS in the pre-operative phase was 38.4 (SD=36.1) days. More than half (52.4%, n = 55/105) of the cardiac patients had a pre-operative hospital LOS above 28 days with (14%, n=15/105) operated on within one week (1-8 days) of their hospital admission (Figure 3). Post-operative LOS was divided into ICU LOS and ward LOS. The mean ICU LOS was 6.4 (SD=5.3) days. The majority of the cardiac patients (85.4%, n=88/103) stayed in ICU for a period of 1-8 days (Figure 3). Only 2% (n=2/103) of the cardiac surgery patients had prolonged ICU stay of more than 28days (Figure 3), 36days being the maximum ICU LOS. The mean ward LOS following cardiac surgery was 12.2 (SD= 7.8) days. A third (33%, n=31/95) of the cardiac surgery patients had a ward LOS of 8 days while 49.5% (n=47/95) of the cardiac surgery patients had a ward LOS of 9 to 20 days (Figure 3).

### Outcome of management

In this study, the outcome of management is defined as the number of patients who developed post-operative complications, the type of complications and mortality. A total of 20.4% (n=21/103) developed post-operative complications. The post-operative complications noted were based on what was documented in the patient medical folders by the doctor. The documented and notable type of post-operative complications included cardiac arrest, pneumonia and lung collapse (Table 1), the last two being of interest to physiotherapists. Other pulmonary complications such as respiratory failure with ventilatory support >48 hours acute lung injury (ALI) including aspiration pneumonitis, transfusion-related ALI (TRALI), acute respiratory distress syndrome (ARDS) and pulmonary embolism were not documented in the 105 records reviewed.

**Table 1:** Distribution of the type of post-operative of complications (n=21)

Type of complication	Frequency (n)	Percentage (%)
Cardiac Arrest	7	33.3
Pneumonia	4	19.0
Sceptic Wound	3	14.3
Ascites	3	14.3
Hemiparesis	2	9.5
Endocarditis	1	4.8
Lung Collapse	1	4.8

A total of 11 deaths were recorded. These 11 cases had a mean LOS of 9.4 (SD=12.7) days. Two (18.2%) cardiac patients died whilst still in theatre. The majority of deaths were in ICU (72.7%, n=8/11) and only 9.1% (n=1/11) died in the ward. A summary of the outcome of all (n=105) cardiac surgery patients is also shown in Table 2. A total of 89.5% (n=94/105) of patients were discharged home.

**Table 2:** Outcome following cardiac surgery (n=105)

Patients outcome	Frequency (n)	Percentage (%)
Discharged home	94	89.5
Died in the theatre	2	1.9
Died in ICU	8	7.6
Died in Ward	1	1.0
Total	105	100

*ICU: Intensive Care Unit		

### Referral for physiotherapy management

Referral in this study was defined as a formal written referral from the Cardiac Surgeon. Only 1.0% (n=1/105) of the cardiac patients were referred for physiotherapy management pre-operatively while more than three quarters (77.7%, n=80/103) of the patients were referred for physiotherapy management post-operatively. Most of the cardiac patients were referred for physiotherapy management on the first day post-operative (70.0%, n=56/80) as presented in Table 3. Physiotherapists could also treat patients on instruction or referral from Anaesthesiologists.

**Table 3:** Distribution of the referral for physiotherapy management (n=103)

Patients referred	Frequency (n)	Percentage (%)
**Pre-operative**		
Yes	1	1.0
No	104	99.0
Post-operative*		
Yes	80	77.7
No	23	22.3
Number of Days Referred Post-operative*		
First Day	56	70.0
Second Day	16	20.0
Third Day	4	5.0
After Third Day	4	3.0

* n=103 as 2 patients died in theatre.

## Discussion

The study identified 105 cardiac cases over the four-year study period of interest with more than half of the cases being female who were young. The main reason for cardiac surgery was due to Rheumatic Heart Disease. Cardiac arrest, pneumonia and lung abscess were the most prevalent complications documented post-operatively. The majority of deaths occurred in the ICU. Referral to physiotherapy was mainly post-operative via written referral from the Cardiac Surgeon with only one percent being referred pre-operatively for physiotherapy intervention.

A decrease of annual cardiac surgeries from 48.6% in 2010 to 10.5% in 2013 was observed in this study. Although this study only focused on patients above 18 years of age, there is still an overall significant decrease in cardiac surgeries when compared to previous studies which recorded 105 procedures in one year^12^,^22^. These challenges are common in low and middle-income countries as they struggle to establish and maintain cardiothoracic centres^3^,^23^,^24^. According to the World Surgical Association^2^ cardiac surgery is an expensive procedure requiring many disposable consumables which are only imported from developed countries. In addition, cardiac surgery utilizes a huge number of resources and therefore keeping a cardiac centre operational requires financial stability and support from both the institute and the government at large. Politics is a further challenge to health care in developing countries. Several researchers have asserted that political instability of a country and the politics within the working environment can easily ruin the achievement which has been obtained in the area of cardiothoracic surgery^9^,^11^,^23^,^25^,^26^. It is not easy to pinpoint how politics in Tanzania has impacted the field of cardiothoracic surgery, though it can be said that political will lacks and does exist from top down or bottom up especially when individuals lobby for change^25^, ^27^-^29^.

The present study has shown that, patients waited on average 38.35 days for surgery as opposed to an average of 8 days reported in Senegal^30^. The longer pre-operatively stay of the majority of patients in this study can be associated to the lack of consumables and limited number of cardiothoracic surgeons in the unit. At the time of the study and from the primary researcher's [ARM] experience of having worked in the cardiac unit in MNH, the long pre-operative length of stay of patients were affected by the lengthy waiting time for equipment and consumables procured by the hospital from suppliers and the need to bring in expert staff from abroad to assist with the cardiac surgeries. Expert medical staff would also only come when there were more than one or two cardiac surgery cases to be operated on to reduce costs of travelling for one patient at a time. It has been observed that a longer pre-operative hospital stay is common in most developing countries and it seems that this will continue due to the lack of consumables, equipment, shortage of staff and financial instability^27^ from which Tanzania is not exempted. At the MNH cardiac unit, patients were first attended to by a Cardiologist who does all investigations to identify the magnitude of the ailment. If the condition required surgery, the patient was referred to the cardiac panel which involves Cardiac Surgeons and other members who discussed the patient before scheduling for surgery. The latter such as awaiting results from medical investigations and awaiting decisions from the cardiac panel regarding surgery could be considered as another set of factors prolonging pre-operative length of stay for patients reviewed in this study. This longer waiting period has a negative impact on the life of patients^10^,^31^. It has been reported that a significant number of patients die in the course of waiting for the life-saving procedure^10^,^31^. This prolonged waiting period could also affect the individual's quality of life and psychological well-being^10^,^31^. It has been reported that high levels of anxiety and stress might be experienced by patients awaiting cardiac surgery due to concern, fear and the uncertainty of the outcome of the surgery^32^,^33^. During this period, pre-operative management could include education about the surgery, expectations after the surgery and physiotherapy such as breathing and general mobility and resistance exercises to maintain lung volume, joint range of motion and muscle strength.^14^, ^34^,^35^

Physiotherapy involvement in cardiac surgery has been confirmed to facilitate quick recovery and promote functional recovery^35^,^36^,^37^. The basis for the involvement of physiotherapy prior to cardiac surgery is to reduce the chances of the development of post-operative respiratory complications^16^. Physiotherapists are not necessarily being able to assert their first line practitioner status in Tanzania^20^ particularly in the cardiac intensive care unit and their involvement in the management of patients undergoing cardiac surgery is dependent on the referrals from cardiothoracic surgeons^21^. Only 1.0 % of patients in this study were referred to physiotherapy pre-operatively and 77.7% of all adult cardiac patients operated on were referred post-operatively in this study. However, the pre-operative and post-operative referral to cardiac rehabilitation is an issue in most countries including developed countries^38^,^39^. As a result, this service is under-utilised, which results in diminishing the effect of the intervention or the outcome of the procedure performed.

In this study, pneumonia and lung collapse were some of the notable documented complications post cardiac surgery. It is known that physiotherapy is used before and after cardiac surgery and that it is thought to be beneficial in reducing the risk of pulmonary complications such as pneumonia and lung collapse^35^. Early mobilisation and breathing activities have been proven to reduce pulmonary complications and improve outcomes including reducing ventilation time and length of stay in general^35^. However, a lack of evidence exists within this regard especially following cardiac surgery and a need for evidence-based interventions is urgently needed to assist physiotherapists in advocating their role in the management of cardiac surgery patients particularly in Tanzania. It is clear from this study that there is a lack of pre-operative physiotherapy in cardiac patients that may be due to a lack of evidence of the benefits of pre-operative physiotherapy and a lack of awareness of cardiac surgeons of these benefits and need for pre-operative referral.

While the study is novel and provides the most current evidence of the profile of cardiac surgery patients and referral to physiotherapy in Tanzania, there are a few limitations of the study. With regards to data collection, there is one main health record at MNH which keeps all folders. However, the Cardiac Unit, paediatrics and out-patients' have their Health Record Units within their complexes. It was a challenge to trace and access some patient medical folders, as the patients attended different clinics using the same medical folder used in the Cardiac Unit. While the latter was a challenge, the primary researcher [ARM] was able to retrieve all the medical records of the patients admitted for cardiac surgery during the study period investigated and thus there were no missing records. In addition, however, while we were able to extract all the relevant information that addressed our study outcomes, we considered the reliance on the doctors' documentation in particular the post-operative complications as a limitation that may affect our results. Another possible limitation to our findings based on the experience of the primary researcher [ARM] who had previously worked in the cardiac unit is the small percentage of pre-operative referrals to physiotherapy that could be due to the doctor not documenting this referral in the patients' medical records although having referred the patient verbally during the multidisciplinary cardiac unit team ward round. The sample of this study was conveniently selected from one hospital in Tanzania and the sample size was relatively small. Thus, generalization of the findings to other Cardiac Centers is limited. Causal inferences cannot be made due to the limited data. The profile of patients and referrals to physiotherapy care on the post-operative management will not necessarily continue to be the same. Therefore, caution should be exercised in interpreting the results in the absence of longitudinal data. Also, caution should be observed when direct or parallel comparison of this study is made with other studies carried out elsewhere in the world, due to different environmental, sampling, and methodological variations.

## Conclusion

Cardiac surgery teams at MNH need to find methods to maintain consistency in the number of surgeries that can be performed annually to cater for the increasing incidence of cardiovascular disease and improve on the pre-operative waiting time. Physiotherapists in the hospital and Tanzania in general need to provide evidence for the efficacy of pre- and post-operative physiotherapy pre and post cardiac surgery and create awareness of pre-operative physiotherapy benefits amongst the cardiac surgeons and rest of the cardiac team to improve referral. While the study provides evidence for the referral to physiotherapy post-operatively, the frequency and type of treatments provided post cardiac surgery are not known and may provide an understanding of the role and practices of physiotherapists in the post-operative phase of cardiac surgery patients in Tanzania and needs assessment.
